# MRI-derived deep-feature model for differentiating stage I endometrial carcinoma from atypical endometrial hyperplasia: a two-center retrospective study

**DOI:** 10.3389/fonc.2026.1943943

**Published:** 2026-09-14

**Authors:** Danhong Chai, Fenghua Ma, Yan Chen, Yapei Zhong, Guofu Zhang, Zhaoxia Qian, Jinwei Qiang

**Affiliations:** 1 Radiology Department, Jinshan Hospital of Fudan University, Shanghai, China; 2 Obstetrics and Gynecology Hospital of Fudan University, Shanghai, China; 3 Radiology Department, Shanghai Jiao Tong University School of Medicine Affiliated International Peace Maternal and Child Health Hospital, Shanghai, China

**Keywords:** atypical endometrial hyperplasia, deep learning, endometrial carcinoma, magnetic resonance imaging, radiomics

## Abstract

**Background:**

Differentiating stage I endometrial carcinoma (EC) from atypical endometrial hyperplasia (AEH) before surgery is critical for guiding treatment decisions, yet current magnetic resonance imaging (MRI) assessment is limited by overlapping appearances and inter-observer variability.

**Methods:**

In this retrospective, two-center study, 297 patients (153 stage I EC and 144 AEH) who underwent multiparametric MRI were included. Radiomics features were extracted from T2-weighted imaging with fat saturation (T2WI-FS), diffusion-weighted imaging (DWI), apparent diffusion coefficient (ADC) map, and contrast-enhanced T1-weighted imaging (CE-T1WI) sequences. Feature selections were performed using univariate filtering, correlation analysis, least absolute shrinkage and selection operator (LASSO) and multivariate stepwise regression. A Radiomics model, a Clin+Rad model, and a DenseNet121-derived deep-feature model were constructed and compared for differentiating stage I EC from AEH. Model performance was evaluated using receiver operating characteristic (ROC) curves, calibration curves, decision curve analysis (DCA).

**Results:**

Seven radiomics features and eight deep-learning features were ultimately selected to construct the models. In the external validation cohort, the DenseNet121-derived deep-feature model achieved the numerically highest AUC of 0.854 (95% CI, 0.743-0.965), compared with 0.803 (95% CI, 0.673-0.933) for the Radiomics model and 0.773 (95% CI, 0.626-0.919) for the Clin+Rad model, although the pairwise differences in AUC were not statistically significant. In addition, the DenseNet121-derived model showed a relatively greater net clinical benefit at threshold probabilities of approximately 0.15-0.80. SHAP analysis indicated that several influential deep features were derived from ADC images, suggesting that ADC-derived features contributed substantially to the model predictions.

**Conclusions:**

The DenseNet121-derived model achieved the numerically highest AUC in the external validation cohort, although the pairwise differences were not statistically significant, indicating its potential as a non-invasive tool to support individualized decision-making.

## Introduction

Endometrial cancer (EC) is one of the most common gynecologic malignancies worldwide, with its incidence increasing steadily over recent decades (1, 2). This growing disease burden has been associated with population aging and the increasing prevalence of obesity and other metabolic risk factors (3). According to the International Federation of Gynecology and Obstetrics (FIGO), the 5-year survival rate exceeds 95% for stage I EC but drops to below 20% for stage IV disease (4), underscoring the importance of early and accurate diagnosis. Atypical endometrial hyperplasia (AEH) is widely regarded as a precursor to EC (5). Dilatation and Curettage (D&C) is commonly used for diagnosis. However, the procedure is performed blindly, it is susceptible to sampling errors and may miss focal lesions (6). Studies have shown that 37%–43% of patients diagnosed with AEH preoperatively are ultimately found to have EC after hysterectomy (7, 8). Nevertheless, up to now, reliable non-invasive tools for differentiating EC from AEH remain lacking (9).

MRI offers a non-invasive approach for evaluating endometrial abnormalities (10). However, diagnostic accuracy is hampered by physiological variations and the marked overlap in imaging characteristics across endometrial lesions. (11–14). For example, AEH frequently demonstrate signal intensity similar to normal endometrium on T2WI (15). Moreover, MRI interpretation depends heavily on radiologist expertise, with reported accuracies ranging from 60% to 75% (14). Therefore, a non-invasive adjunctive tool would be of significant clinical value for preoperative differentiation and treatment decision-making.

Recent advances in radiomics and convolutional neural networks (CNNs) have created new possibilities for image-based diagnosis. Radiomics extracts quantitative imaging features to characterize disease phenotypes and assist in classification (16). CNNs automatically learn hierarchical imaging representations to support feature extraction and classification (14, 17). Besides, CNNs capture subtle spatial features and have demonstrated strong performance across tasks (18, 19). Their effectiveness has been validated across multiple clinical applications, including breast cancer detection (20), cervical cancer segmentation (21), and gastric disease classification (22).

Recently, several studies have explored non-invasive differentiation of endometrial diseases. Zhang et al. developed an MRI-based radiomics model to differentiate EC from AEH, reporting an accuracy of 93.2%; however, its single-center design and limited sample size restrict its generalizability (23). Takahashi et al. proposed a hysteroscopy-based machine learning model that achieved accuracies of 85% for EC and 100% for AEH (24). To our knowledge, few studies have specifically investigated mp-MRI based deep-learning approaches for the preoperative differentiation of stage I EC from AEH.

## Materials and methods

### Data collection

This retrospective study was approved by the Review Board of Center A and Center B, both of which waived the requirement for written informed consent. A total of 648 patients from Center A (January 2021–December 2023), and 105 patients from the Center B (January 2020–December 2023), were initially screened. All included patients underwent MRI examinations before hysteroscopic biopsy or hysterectomy. The interval between MRI and pathological sampling did not exceed 7 days. All patients had undergone diagnostic curettage or hysterectomy, and all diagnoses of endometrial-related diseases were pathologically confirmed.

The inclusion criteria were as follows: (1) Histopathologically confirmed stage I EC or AEH; (2) Preoperative multiparametric pelvic MRI, including axial T2-weighted imaging with fat saturation (T2WI-FS), diffusion-weighted imaging (DWI), apparent diffusion coefficient (ADC) mapping, and contrast-enhanced T1-weighted imaging (CE-T1WI); and (3) No history of prior radiotherapy, chemotherapy, hormonal therapy, or comorbid gynecological tumors. Exclusion criteria were: (1) Lesions too small for reliable delineation (n=78); (2) Poor-quality images or severe artifacts (n=23); (3) Patients not classified as stage I EC (n=64); (4) Incomplete imaging sequences or export failure (n=153); and (5) Presence of other concurrent malignancies (n=138). Ultimately, 297 patients met the eligibility criteria, including 251 patients from Center A (124 stage I EC and 127 AEH) and 46 patients from Center B (29 stage I EC and 17 AEH). The 251 patients from Center A were randomly divided into a training cohort (n = 176) and an internal testing cohort (n = 75) at a ratio of approximately 7:3 using stratified random sampling according to disease status. The overall workflow is illustrated in **Figure 1**.

**Figure 1:**
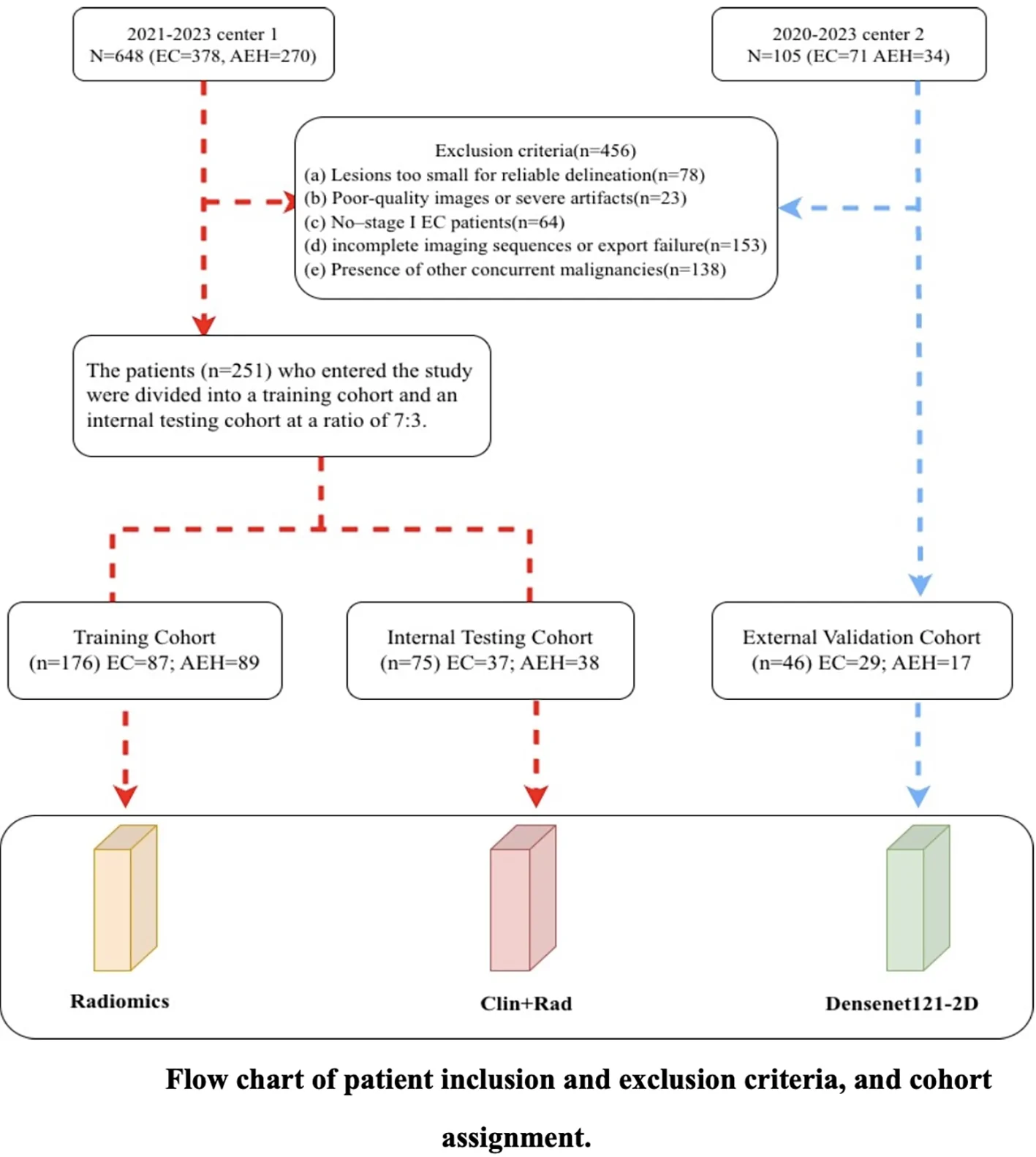
Flowchart of inclusion and exclusion criteria.

### MRI acquisition

MRI examinations were performed using a 1.5-T scanner (Avanto and; Siemens, Erlangen, Germany) equipped with a phased-array abdominal coil. The detailed MRI acquisition parameters are provided in **Table 1**. All patients were scanned in the supine position under free-breathing conditions. The routine MRI protocol included axial T2WI-FS, DWI with corresponding ADC maps, and late arterial phase CE-T1WI. DWI was performed with b-values of 1000 s/mm^2^ or 800 s/mm^2^. In addition, it was performed using a single-shot echo-planar imaging sequence with spectral attenuated inversion recovery (SPAIR) fat suppression. CE-T1WI images were acquired approximately 90 seconds after intravenous administration of gadopentetate dimeglumine (0.2 ml/kg) at an injection rate of 2–3 mL/s. A representative case is shown in **Supplementary Figure 1**.

**Table 1:** MRI equipment, scanning protocols, and parameters at different centers.

Center	MRI field strength / model / brand	MRI sequence	b s/mm^2^	Sequence type	TR ms	TE ms	matrix	slice thickness mm	GAP mm
A	1.5T Avanto Siemens	T2WI-FS		SE	4000-6500	83	256×256	4.5	2
		DWI	0, 1000	EPI	2400-3300	83	176×176	5	1.5-2
		CE-T1WI		VIBE	4.9	2.4	256×256	5	0
B	1.5T MAGNETOM Aera Siemens	T2WI-FS		TSE	4200-5200	82	640×640	4.5-5	0.9-1
		DWI	0, 800	EPI	5100	80	336×336	5	0.8
		CE-T1WI		VIBE	4.5	2.2	640×640	3	0

ADC maps were automatically generated from the acquired DWI data and were therefore not treated as independently acquired MRI sequences.

(ADC, apparent diffusion coefficient mapping; CE-T1WI, contrast-enhanced T1-weighted imaging; DWI, diffusion-weighted imaging; MRI, magnetic resonance imaging; T2WI-FS, T2-weighted imaging with fat-saturation; TE, echo time; TR, repetition time; SE, spin echo; TSE, turbo spin echo; EPI, echo planar imaging; VIBE, volume interpolated breath-hold examination).

### Clinical data analysis

Preoperative clinicopathological data were collected, including age, body mass index (BMI), obstetric history, menopausal status, hypertension, diabetes, hyperlipidemia, and tumor markers such as CA125 and CA199. Clinical variables with less than 20% missingness were imputed using interpolation. The imputation procedure and associated parameters were derived exclusively from the training cohort and applied unchanged to the internal testing and external validation cohorts. Variables with more than 20% missingness were excluded from model construction. Potential clinical predictors were initially screened using univariate analysis (*p* < 0.05) and were subsequently evaluated with multivariate stepwise logistic regression. Ultimately, age and menopausal were retained as the two independent clinical predictors for model construction.

### Radiomics feature extraction

All MRI data were imported into ITK-SNAP (version 4.2.0) for processing. Radiologist 1, who had three years of experience in pelvic imaging, independently delineated the tumor boundaries on each axial slices. ROI segmentation was performed separately on each sequence (T2WI, DWI, ADC, and late arterial phase CE-T1WI), with the radiologist blinded to all clinical information and pathological results. (**Supplementary Figure 2**). After 2 months, radiologist 1 and radiologist 2 (with eight years of experience in pelvic imaging) then independently performed a second round of segmentation on a randomly selected subset of 50 patients. During this process, they carefully excluded endometrial cavity fluid, hematoma, and adjacent normal myometrium, while allowing necrotic, hemorrhagic, or cystic components within the lesion to be included. Feature stability was calculated using both inter- and intraclass correlation coefficients (ICC), and only features with ICC value greater than 0.8 were retained.

Segmentation of the volume of interest (VOI) encompassing the entire tumor was then performed. Before feature extraction, all original images were resampled to a voxel size of 1.5 × 1.5 × 1.5 mm³ using B-spline interpolation, while nearest-neighbor interpolation was applied to the corresponding segmentation masks. A total of 6,752 radiomic features were extracted using PyRadiomics version 2.2.0. Intensity values were standardized using z-score normalization. Gray-level discretization was performed using a fixed number of bins, with 5 bins for T2WI, DWI, and late arterial phase CE-T1WI images and 50 bins for ADC images. Features were extracted from the original images and filtered images, including Wavelet, Laplacian of Gaussian (LoG), SquareRoot, Logarithm, Exponential, Gradient, and Local Binary Pattern (LBP) filters. Eight wavelet decompositions and three LBP3D parameter settings were applied. B-spline interpolation was used for image resampling, whereas nearest-neighbor interpolation was applied for mask resampling. All feature extraction procedures followed IBSI-compliant definitions. Detailed feature extraction parameters are provided in **Supplementary Table 1**.

### DL feature extraction

A two-dimensional, slice-based strategy was adopted for deep feature extraction. Specifically, for each MRI sequence (T2WI-FS, DWI, ADC, and late arterial phase CE-T1WI), the axial slice containing the largest cross-sectional tumor area was selected based on radiologist-annotated masks. The selected slice was then cropped using a bounding box tightly enclosing the lesion to remove irrelevant background information. Original images were resampled to a voxel size of 1.5 × 1.5 × 1.5 mm³ to standardize spatial resolution across subjects. The cropped images were subsequently resized to 224 × 224 pixels and intensity-normalized using z-score normalization. Because the ImageNet-pretrained DenseNet121 requires three-channel input, each single-channel MRI slice was replicated across the three channels before being independently input into the network. A DenseNet121-2D CNN pre-trained on ImageNet was used as a frozen feature extractor without fine-tuning. The outputs of the penultimate layer were extracted as deep features, yielding 1,024 features for each MRI sequence and a total of 4,096 deep features across the four MRI sequences. A fixed random seed of 697 was used throughout the analysis to ensure reproducibility.

### Feature selection, and modeling

For both the radiomics and deep-feature models, redundant or irrelevant features were removed using the same sequential feature-selection strategy: univariate filtering, correlation analysis, least absolute shrinkage and selection operator (LASSO) regression, and multivariate stepwise logistic regression. Univariate filtering was performed using the Mann–Whitney U test (p < 0.01), followed by correlation analysis. LASSO regression with 5-fold cross-validation was used to determine the optimal regularization parameter (λ min). The penalty parameter (λ) was determined using the minimum cross-validation error criterion (λ min), and features with nonzero coefficients at the selected λ were retained (**Supplementary Figure 3**). Following LASSO-based dimensionality reduction, multivariate stepwise logistic regression was applied to the retained features to obtain a more parsimonious final feature set and further reduce residual redundancy among candidate predictors. All supervised feature-selection procedures were performed exclusively within the training cohort; the internal testing and external validation cohorts were not involved in feature selection or model development.

For the Radiomics model, the 6,752 initial features were reduced to 1,293 after univariate filtering, 104 after correlation analysis, 11 nonzero features after LASSO regression, and 7 final features after multivariate stepwise logistic regression. The seven retained features were used to construct the Radiomics model.

For the DenseNet121-derived deep-feature model, the 4,096 initial deep features were reduced to 750 after univariate filtering, 24 after correlation analysis, 11 nonzero features after LASSO regression, and 8 final features after multivariate stepwise logistic regression. The final eight selected deep features comprised two derived from T2WI-FS, one from DWI, and five from ADC. The eight retained features were entered into a standard logistic regression classifier without regularization or class weighting to construct the DenseNet121-derived deep-feature model.

A combined clinical–radiomics (Clin+Rad) model was then generated by integrating independent clinical predictors. After combining the seven selected radiomics features with the two independent clinical predictors, Spearman correlation analysis was further performed to identify highly correlated predictors (|r| ≥ 0.90) and reduce redundancy. After correlation-based filtering, six predictors were retained to construct the final Clin+Rad model. The detailed experimental workflow is illustrated in **Figure 2**. The regression coefficients and complete equations of the final Radiomics, DenseNet121-derived deep-feature, Clinical, and Clin+Rad models are provided in **Supplementary Table 2**.

**Figure 2:**
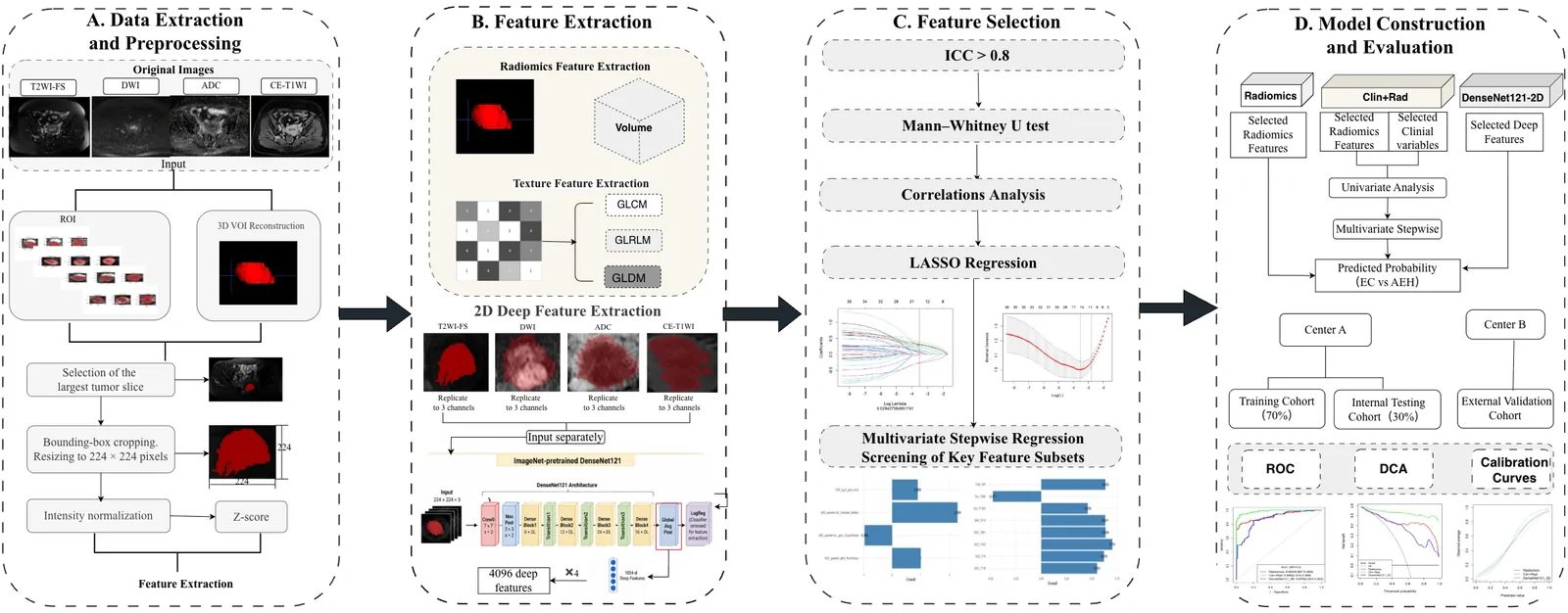
The workflow of model diagram.

### Statistical analysis

Statistical analyses were performed using R software (version 4.4.0). Categorical variables were compared using chi-square test or Fisher’s exact test. Continuous variables were first assessed for normality, and analyzed using either the independent samples Mann–Whitney U test, as appropriate. Feature dimensionality reduction proceeded sequentially through inter- and intra-observer ICC assessment, univariate filtering, correlation analysis, LASSO regression, and multivariate stepwise regression. The extracted DenseNet121-derived deep-features were incorporated into a logistic regression classifier to construct the DL-based model. Model performance was assessed using the area under the curve (AUC) of receiver operating characteristic (ROC) and calibration curves and decision curve analysis (DCA). Nonparametric bootstrap resampling with 200 iterations was used to estimate 95% confidence intervals (CIs) for performance metrics, including accuracy, sensitivity, specificity, positive predictive value (PPV), negative predictive value (NPV), AUC, and calibration measures. The optimal probability threshold for each model was determined exclusively in the training cohort using the Youden index and subsequently fixed for evaluation in the internal testing and external validation cohorts. The resulting thresholds were 0.555 for the Radiomics model, 0.495 for the Clin+Rad model, and 0.563 for the DenseNet121-derived deep-feature model.

Calibration was evaluated using calibration curves, calibration intercept, calibration slope, and Brier score, with 95% CIs calculated using the aforementioned bootstrap procedure. Confusion matrices (CMs) were defined as 2 × 2 contingency tables and used to derive true-positive (TP), false-positive (FP), false-negative (FN), and true-negative (TN) counts. Differences in AUCs between models were assessed using DeLong’s test. All statistical tests were two-sided, and P < 0.05 was considered statistically significant.

## Results

### Patient characteristics

A total of 297 patients were included, comprising 153 patients with stage I EC and 144 patients with AEH. The median age was 48 years (Q1-Q3: 34–58 years). Baseline characteristics of patient with stage I EC or AEH in the training, internal testing, and external validation cohorts are summarized in **Table 2**.

**Table 2:** Comparison of clinical characteristics between EC and AEH patients across cohorts.

Characteristics	N	Overall (N = 297) (144 AEH, 153 EC)	Training (N = 176)		*p*-value	Internal testing (N = 75)		*p*-value	External validation (N = 46)		*p-*value
			AEH 89	EC 87		AEH 38	EC 37		AEH 17	EC 29	
Age, Median [Q1–Q3] ^1^	297	48.0 [34.0,58.0]	34.0 [30.0,40.0]	58.0 [51.0,65.0]	< 0.001	33.5 [30.0,39.0]	56.0 [53.0,66.0]	< 0.001	50.0 [45.0,58.0]	55.0 [48.0,59.0]	0.322
Obstetric history (N/Y) ^2,3^	293	99/194	58/28	6/80	< 0.001	22/16	6/31	< 0.001	4/13	3/26	0.184
Menopause (N/Y) ^2,3^	292	175/117	84/2	21/65	< 0.001	37/1	7/29	< 0.001	12/5	14/15	0.141
Menopause Age, Median [Q1–Q3] ^1^	117	50.0 [49.0,53.0]	50.0 [49.0,51.0]	50.0 [49.0,52.0]	0.837	50.0 [50.0,50.0]	51.0 [49.0,53.0]	0.683			0.948
BMI, Median [Q1–Q3] ^1^	211	24.8 [22.1,28.3]	26.1 [22.0,30.3]	24.3 [22.2,27.4]	0.130	25.4 [22.0,28.2]	25.3 [22.3,27.5]	0.423	NA [NA,NA]	NA [NA,NA]	
Hypertension (N/Y) ^2,3^	295	218/77	78/10	56/30	< 0.001	35/3	23/14	0.002	11/6	15/14	0.391
Diabetes (N/Y)^2,3^	295	254/41	81/7	66/20	0.005	35/3	30/7	0.191			0.619
Hyperlipidemia (N/Y) ^2,3^	295	281/14	85/3	81/5	0.494	37/1	33/4	0.200	16/1	29/0	0.370
CA125 (-/+)^2,4^	270	239/31	74/7	75/9	0.653	31/4	26/7	0.274	12/1	21/3	1.000
CA199 (-/+)^2,4^	165	137/28	23/1	53/14	0.105	11/1	18/8	0.223	10/2	22/2	0.588
HPV (N/Y) ^2,3^	139	126/13	36/5	47/3	0.460	13/1	17/3	0.627	3/0	10/1	1.000

^1^ Wilcoxon rank sum test; ^2^Pearson's Chi-squared test; Fisher's exact test; ^3^(N/Y) represents no or yes of the corresponding clinical condition; ^4^(-/+) represents negative or positive results, respectively.

AEH, atypical endometrial hyperplasia; BMI, body mass index; EC, endometrial carcinoma; HPV, human papillomavirus; NA, not available.

### Model performance

The diagnostic performances of the models for differentiating stage I EC from AEH are summarized in **Table 3**. The corresponding CMs are presented in **Figure 3**. ROC curve (**Figures 4A–C**) analysis demonstrated that although the DenseNet121-derived deep-feature model showed slightly lower AUCs than the Radiomics and Clin+Rad models in the training cohort (0.882 vs. 0.913 vs. 0.973) and the internal testing cohort (0.821 vs. 0.919 vs. 0.979), it outperformed both models in the external validation cohort, achieving an AUC of 0.854, compared with 0.803 for the Radiomics model and 0.773 for the Clin+Rad model. In the external validation cohort, the DenseNet121-2D model achieved a sensitivity of 55.2% and a specificity of 94.1%. The specificity was comparable to that of the Radiomics model (94.1%) and higher than that of the Clin+Rad model (88.2%), while the sensitivity was lower than that of the Clin+Rad model (58.6%) but higher than that of the Radiomics model (31.0%).

**Table 3:** Predictive performance of different models in the training, internal testing and external validation cohorts.

	AUC	Accuracy (%)	Sensitivity (%)	Specificity (%)	PPV (%)	NPV (%)
Training (n = 176)						
Clinical	0.934 (0.893-0.974)	88.1 (82.3-92.5)	90.8 (82.7-95.9)	85.4 (76.3-92.0)	85.9 (77.0-92.3)	90.5 (82.1-95.8)
Radiomics	0.913 (0.869-0.956)	85.2 (79.1-90.1)	80.5 (70.6-88.2)	89.9 (81.7-95.3)	88.6 (79.5-94.7)	82.5 (73.4-89.4)
Clin+Rad	0.973 (0.953-0.993)	92.6 (87.7-96.0)	89.7 (81.3-95.2)	95.5 (88.9-98.8)	95.1 (88.0-98.7)	90.4 (82.6-95.5)
DenseNet 121-derived model	0.882 (0.831-0.932)	81.8 (75.3-87.2)	77.0 (66.8-85.4)	86.5 (77.6-92.8)	84.8 (75.0-91.9)	79.4 (70.0-86.9)
Internal testing (n = 75)						
Clinical	0.919 (0.846-0.991)	89.3 (80.1-95.3)	86.5 (71.2-95.5)	92.1 (78.6-98.3)	91.4 (76.9-98.2)	87.5 (73.2-95.8)
Radiomics	0.919 (0.860-0.978)	81.3 (70.7-89.4)	78.4 (61.8-90.2)	84.2 (68.7-94.0)	82.9 (66.4-93.4)	80.0 (64.4-90.9)
Clin+Rad	0.979 (0.950-1.000)	93.3 (85.1-97.8)	94.6 (81.8-99.3)	92.1 (78.6-98.3)	92.1 (78.6-98.3)	94.6 (81.8-99.3)
DenseNet 121-derived model	0.821 (0.726-0.915)	72.0 (60.4-81.8)	62.2 (44.8-77.5)	81.6 (65.7-92.3)	76.7 (57.7-90.1)	68.9 (53.4-81.8)
External validation (n = 46)						
Clinical	0.608 (0.415-0.800)	63.0 (47.5-76.8)	86.2 (68.3-96.1)	23.5 (6.8-49.9)	65.8 (48.6-80.4)	50.0 (15.7-84.3)
Radiomics	0.803 (0.673-0.933)	54.3 (39.0-69.1)	31.0 (15.3-50.8)	94.1 (71.3-99.9)	90.0 (55.5-99.7)	44.4 (27.9-61.9)
Clin+Rad	0.773 (0.626-0.919)	69.6 (54.2-82.3)	58.6 (38.9-76.5)	88.2 (63.6-98.5)	89.5 (66.9-98.7)	55.6 (35.3-74.5)
DenseNet 121-derived model	0.854 (0.743-0.965)	69.6 (54.2-82.3)	55.2 (35.7-73.6)	94.1 (71.3-99.9)	94.1 (71.3-99.9)	55.2 (35.7-73.6)

AUC, area under the receiver operating characteristic curve; PPV, positive predictive value; NPV, negative predictive value; Clin+Rad, clinical-radiomics model. Values are presented as estimates with 95% confidence intervals in parentheses. Confidence intervals were calculated using nonparametric bootstrap resampling with 200 iterations.

**Figure 3:**
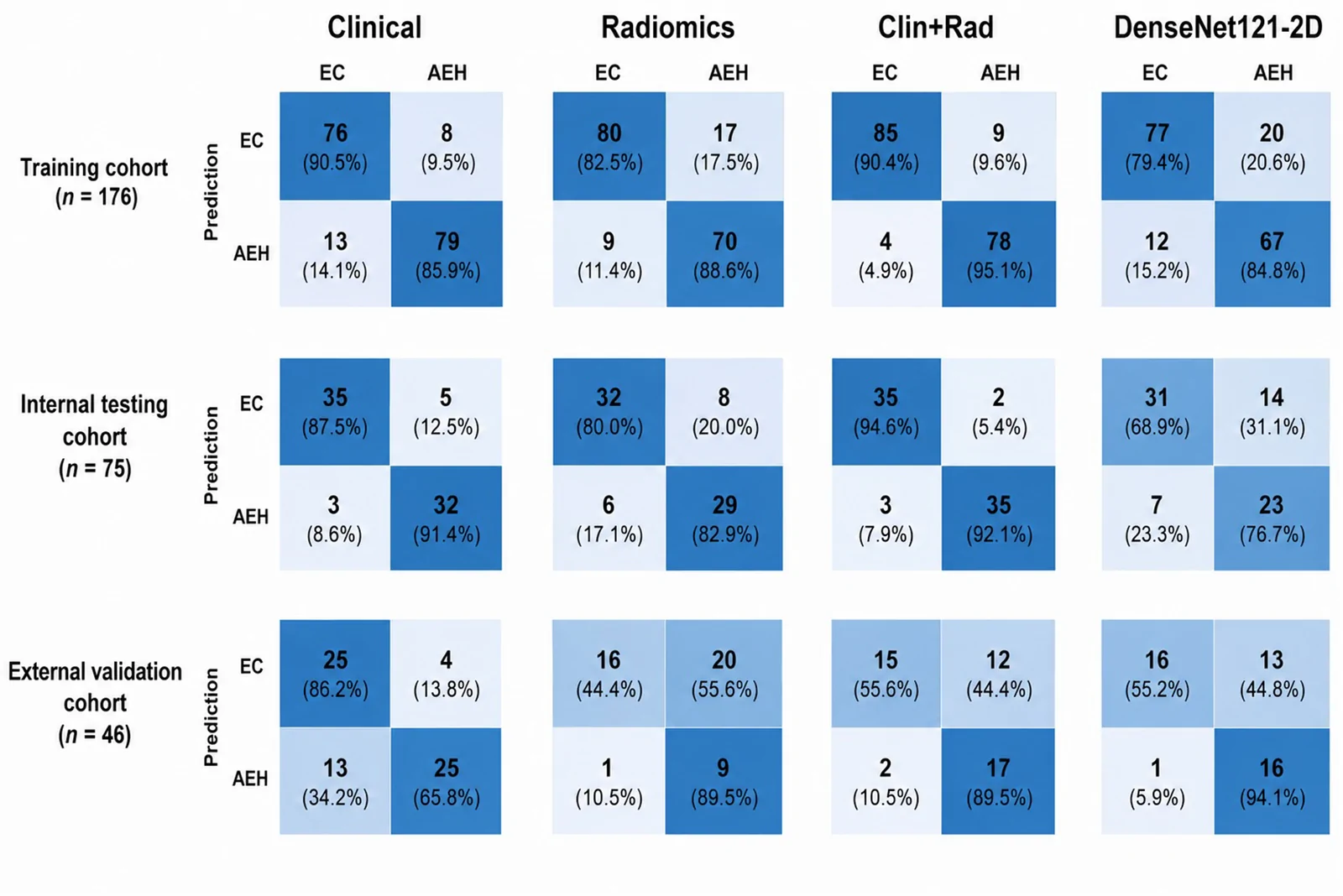
CM for distinguishing EC from AEH across different models. Confusion matrices (CM) for the Clinical, Radiomics, Clin+Rad, and DenseNet121-2D models across the three cohorts. To facilitate comparisons among cohorts with different sample sizes, percentages were calculated by row standardization and are presented alongside the absolute counts in each cell. EC was considered the positive class and AEH was considered the negative class. (CM, Confusion matrix; Clin+Rad, Clinical+Radiomics; AEH, atypical endometrial hyperplasia; EC, endometrial carcinoma).

**Figure 4:**
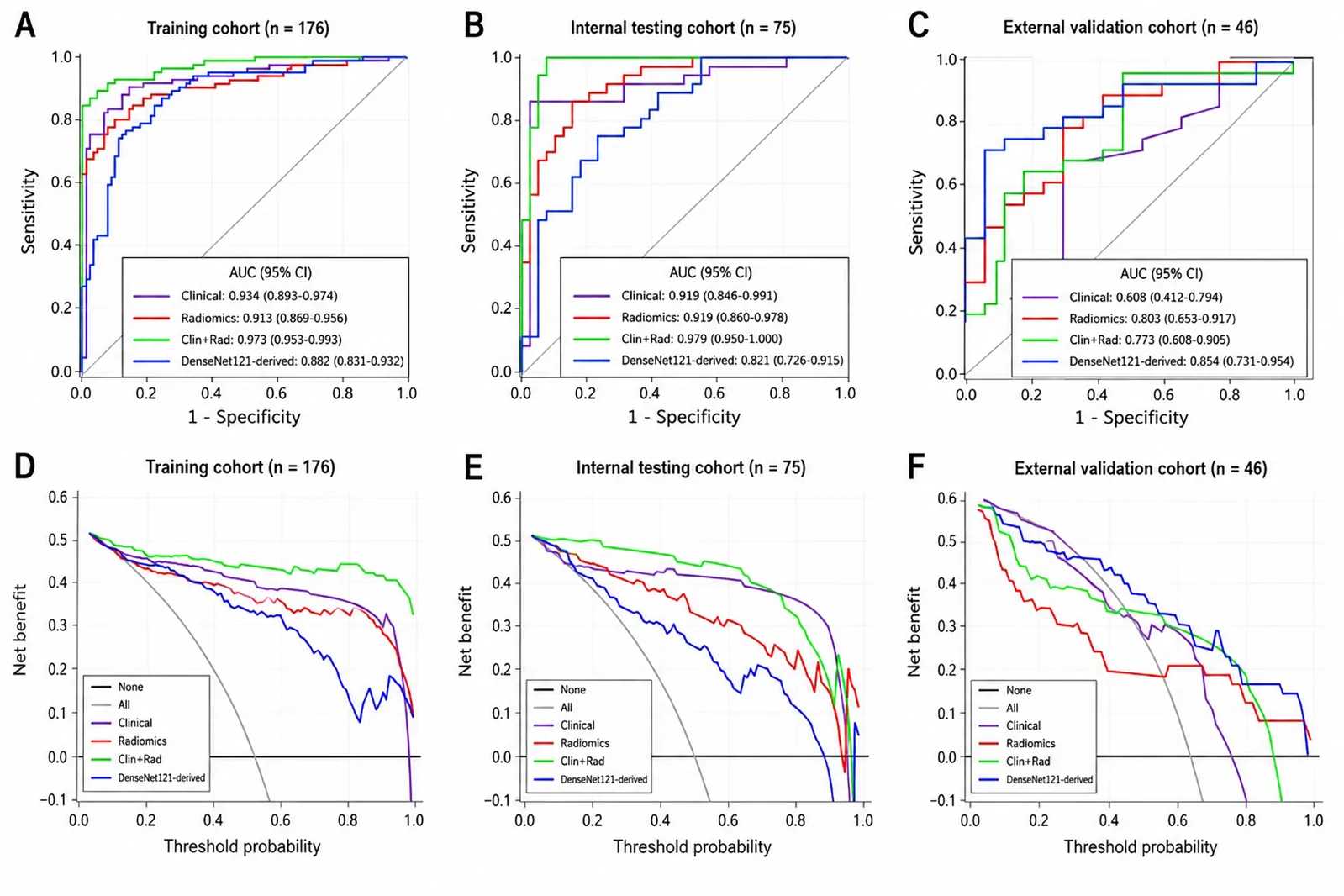
Model performance assessed by ROC and DCA. ROC and DCA plots comparing the diagnostic performance and clinical utility of the Clinical, Radiomics, Clin+Rad, and DenseNet121-derived deep-feature models in the training, internal testing, and external validation cohorts. **(A–C)** illustrate the ROC curves, and **(D–F)** illustrate the corresponding DCA curves for the three cohorts. In the DCA plots, the black horizontal line indicates the strategy of treating no patients, whereas the gray line indicates the strategy of treating all patients. (ROC, receiver operating characteristic; DCA, decision curve analysis).

According to the DeLong test, the Clin+Rad model showed significantly better diagnostic performance than the Radiomics model (P = 0.001) and the DenseNet121-2D model (P < 0.001) in the training cohort, as shown in **Table 4**. However, in the external validation cohort, the DeLong test showed no statistically significant differences in AUC among the models. Nevertheless, additional analyses based on net reclassification improvement (NRI) and integrated discrimination improvement (IDI) revealed that the IDI of DenseNet121-derived deep-feature relative to the Radiomics model remained statistically significant (P = 0.022), whereas the corresponding NRI was not significant. Furthermore, no significant differences in either NRI or IDI were found between DenseNet121-derived deep-feature and the Clin+Rad model. These results indicate that the incremental diagnostic benefit of DenseNet121-derived deep-feature in the external validation cohort was limited, although its potential value should be further investigated in a larger sample. Detailed results are provided in **Supplementary Table 3**.

**Table 4:** DeLong test results for pairwise comparisons of AUCs among different models in different cohorts.

Comparative performance of different models	P value		
	Training	Internal testing	External validation
Radiomics vs Clin+Rad	0.001	0.038	0.668
Radiomics vs DenseNet121-derived model	0.298	0.025	0.447
DenseNet121-derived model vs Clin+Rad	<0.001	0.002	0.315

### Clinical utility and calibration

DCA (**Figures 4D–F**) demonstrated different patterns of net clinical benefit across the three cohorts. The Clin+Rad model generally provided the highest net clinical benefit across most threshold probabilities in the training and internal testing cohorts. In contrast, in the external validation cohort, the DenseNet121-derived deep-feature model provided a relatively greater net clinical benefit at threshold probabilities of approximately 0.15-0.80. As shown in **Figure 5**, calibration analysis showed generally good agreement between the predicted and observed probabilities in the training cohorts, with calibration slopes close to 1.00 and Brier scores ranging from 0.06 to 0.14. In the internal testing cohorts, the calibration curves remained relatively close to the ideal line, with Brier scores ranging from 0.05 to 0.18 and calibration slopes of 0.67-0.91. In the external validation cohorts, greater deviations from the ideal line were observed, with Brier scores ranging from 0.20 to 0.34 and calibration slopes of 0.13-0.84. The corresponding calibration intercepts also varied across the models, reflecting differences in the agreement between predicted probabilities and observed outcomes among the three cohorts.

**Figure 5:**
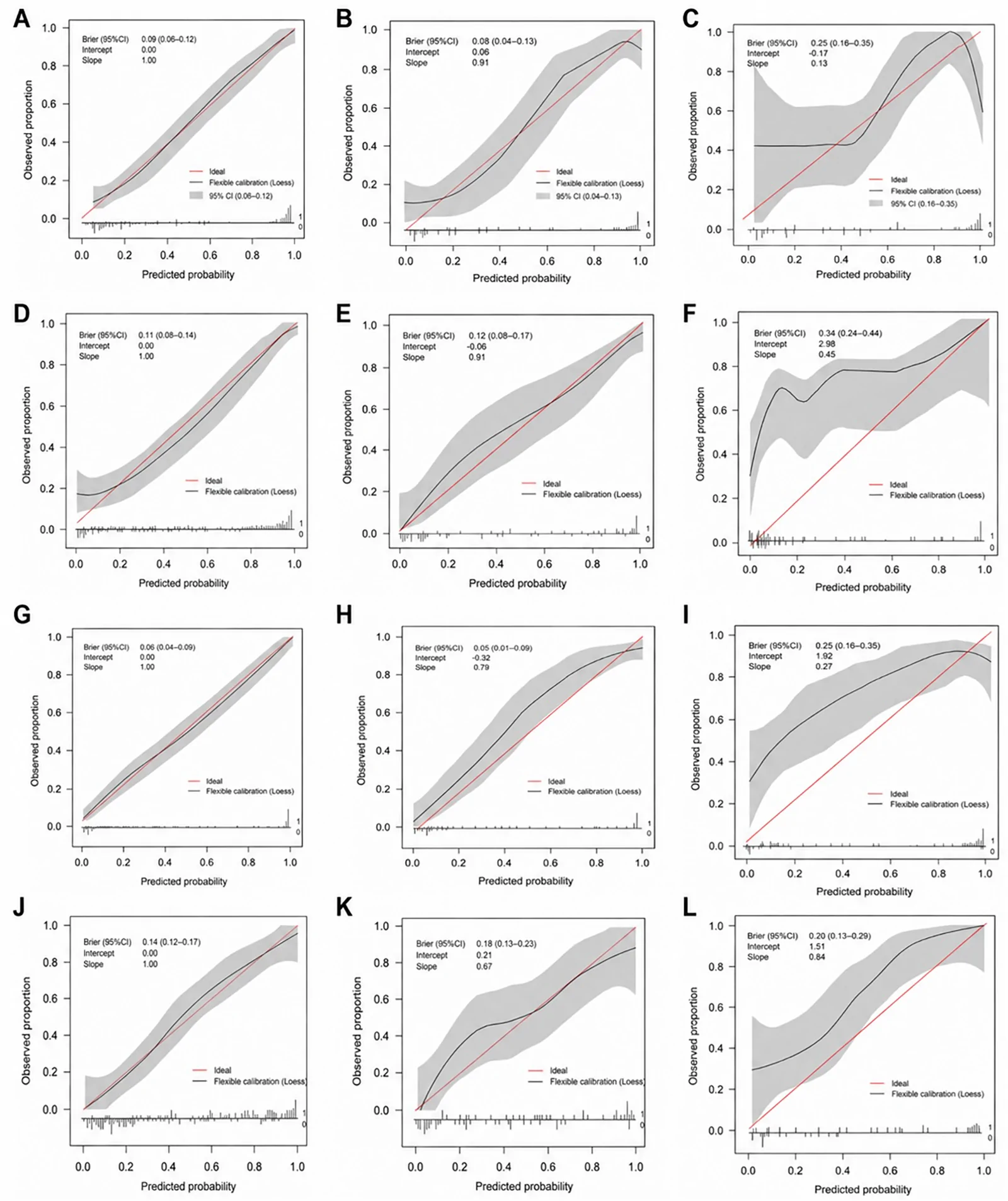
Calibration curves of the Clinical, Radiomics, Clin+Rad, and DenseNet121-derived deep-feature models across different models. Calibration curves of the four predictive models across the training, internal testing, and external validation cohorts. **(A–C)** represent the Clinical model; **(D–F)** represent the Radiomics model; **(G–I)** represent the Clin+Rad model; and **(J–L)** represent the DenseNet121-derived deep-feature model. Within each model, the calibration curves are presented for the training, internal testing, and external validation cohorts, respectively. The red diagonal line represents the ideal calibration line, whereas the black line represents the locally estimated calibration curve generated using locally weighted scatterplot smoothing (LOESS). The gray shaded area indicates the 95% confidence interval. Calibration performance was assessed using the calibration intercept, calibration slope, and Brier score, which are reported within each figure.

### Correlation analysis and model robustness

Pearson correlation analysis was performed to assess the relationships among the final features retained in each model (**Figure 6**). In the radiomics model, most feature pairs showed weak-to-moderate correlations, although a relatively strong positive correlation was observed between two ADC-derived wavelet features (r = 0.78) (**Figure 6A**). In the Clin+Rad model, age and menopausal status were moderately correlated (r = 0.63), while the highest correlation was observed between two ADC-derived radiomic features (r = 0.75) (**Figure 6B**). For the DenseNet121-derived deep-feature model, correlations among the retained deep features were generally low to moderate, with the largest absolute correlation being −0.56 (**Figure 6C**). Overall, no very high pairwise correlations were observed among the final retained features.

**Figure 6:**
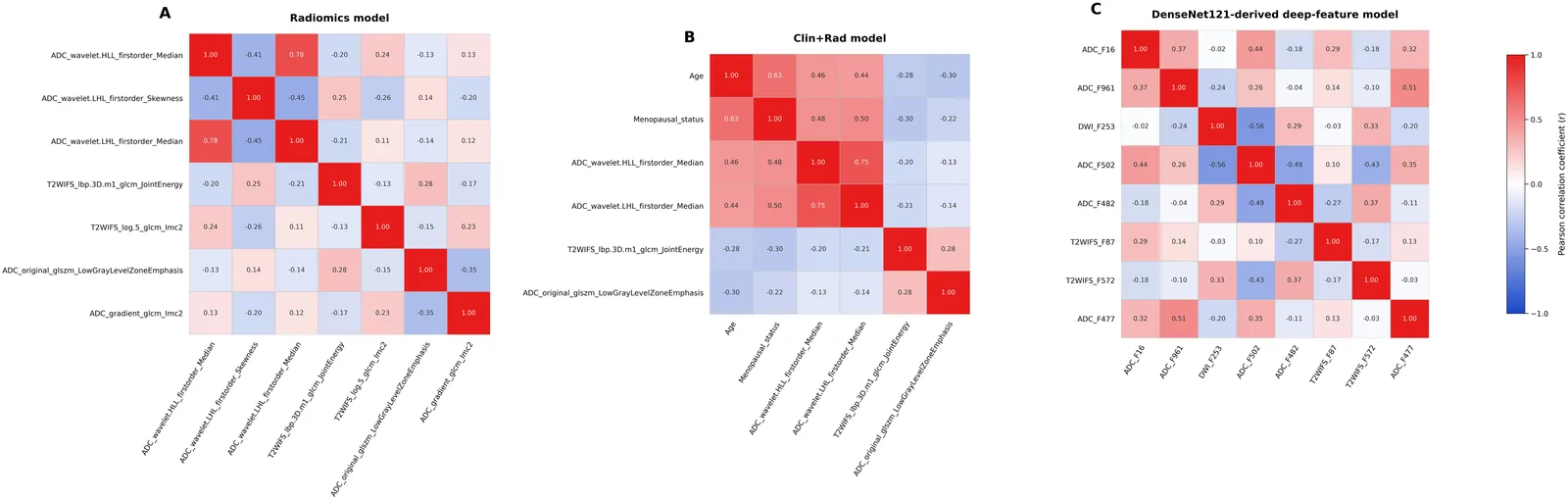
Heatmap of the correlation matrices across multiple feature sets. Pearson correlation matrices illustrating the relationships among the final features retained after multivariate stepwise logistic regression. **(A)** Radiomics model (7 features). **(B)** Clin+Rad model (6 variables, including 2 clinical variables and 4 radiomics features). **(C)** DenseNet121-derived deep-feature model (8 features). The heatmaps display pairwise correlations among model features rather than correlations between individual features and the outcome variable. A Spearman correlation analysis was performed during feature selection to eliminate highly collinear variables before model construction.

### SHAP interpretability analysis

To further evaluate model interpretability, SHAP analysis was applied to the DL model (**Figure 7**). Among all extracted features, the most influential were DWI-F253, ADC-F961, ADC-F502, T2WI-FS-F87, and ADC-F16 in the training cohort. Notably, these same five features remained the top contributors in the external validation cohort, although with varying magnitude. In the internal testing cohort, DWI-F253, T2WI-FS-F87, ADC-F961, and ADC-F502 continued to rank among the most important features. Several influential deep features were derived from ADC images, suggesting that ADC images contributed substantially to model prediction; however, the biological meaning of individual latent features remains uncertain.

**Figure 7:**
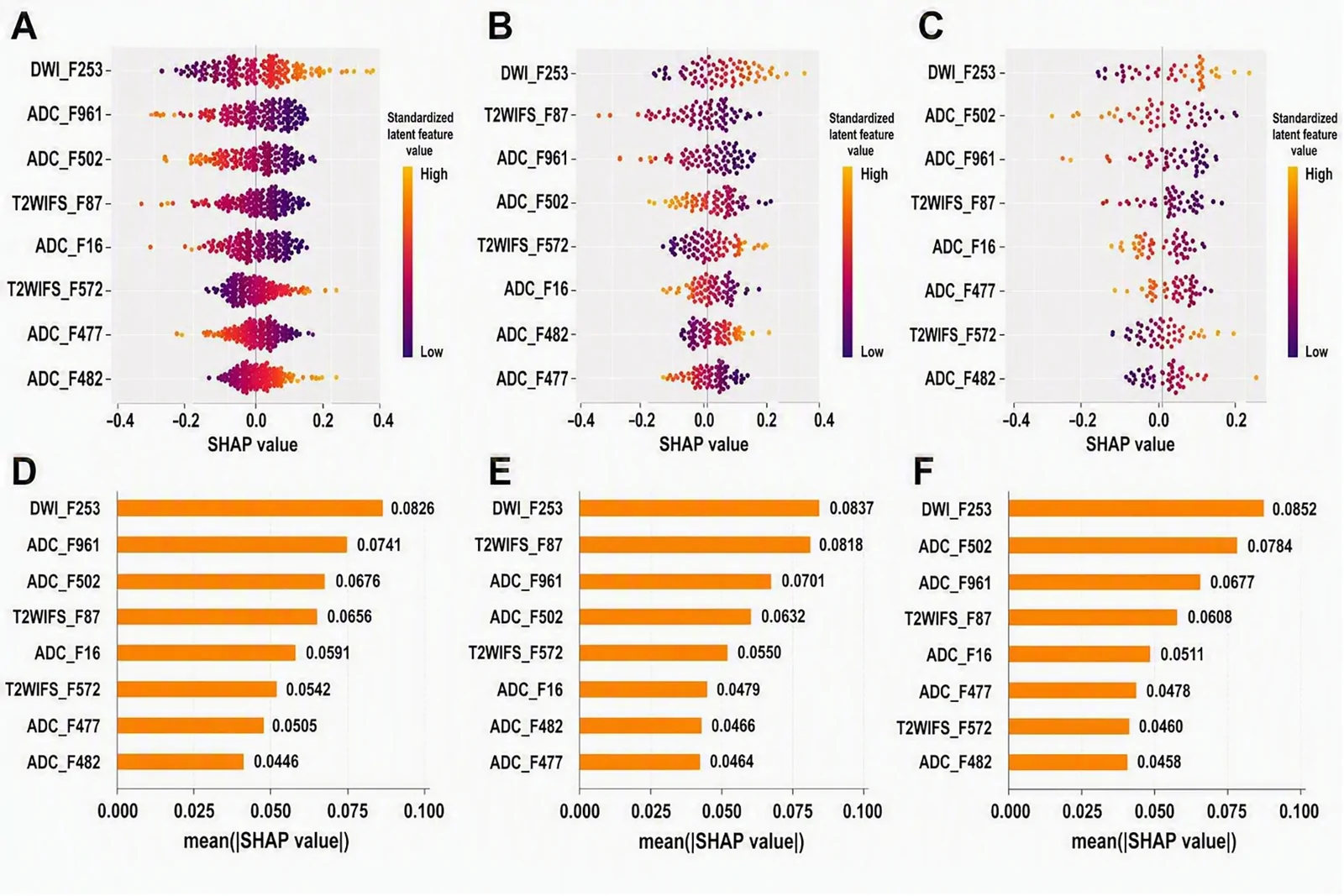
SHAP-based interpretation of the DenseNet121-derived deep-feature model across the training, internal testing, and external validation cohorts. SHAP-based interpretation of the DenseNet121-derived deep-feature model in the training, internal testing, and external validation cohorts. **(A–C)** present SHAP summary plots for the training, internal testing, and external validation cohorts, respectively. Each point represents an individual patient, and the color indicates the standardized value of the corresponding latent feature. Positive SHAP values indicate a greater contribution to the prediction of EC, whereas negative SHAP values indicate a greater contribution to the prediction of AEH. **(D–F)** show the corresponding mean absolute SHAP values used to rank feature importance in the three cohorts. SHAP values were calculated using the Kernel Explainer algorithm and were applied to the final logistic regression classifier constructed from the selected deep features. (SHAP: shapley additive explanations).

## Discussion

In this study, we constructed and validated a DL-based diagnostic model (DenseNet121-derived) that can automatically differentiate stage I endometrial carcinoma (EC) from atypical endometrial hyperplasia (AEH) using datasets from two centers. The model comparable performance in the training cohort (AUC, 0.882) and internal testing cohort (AUC, 0.821). Importantly, in the external validation cohort, the DenseNet121-derived deep-feature model demonstrated satisfactory performance and may provide complementary information to differentiate stage I EC and AEH. This method may help reduce misdiagnosis and improve diagnostic efficiency, and potentially decrease unnecessary surgeries for patients with AEH. Given its underlying architecture, the proposed model could be further extended to support intelligent, image-based auxiliary diagnoses for other diseases. However, the superiority of DenseNet121-derived deep-feature over conventional approaches was not statistically confirmed and requires validation in larger multicenter cohorts.

In the present study, we built both a Radiomics model and a DenseNet121-derived deep-feature model to differentiate patients with stage I EC and AEH. The reliability of image-derived features is critical for building robust radiomics and DL models (25). However, processes such as target segmentation, feature extraction, feature selection, and model construction are inherently subject to inter- and intra-observer variability (26). To address this limitation, manual segmentation was adopted due to the relatively limited sample size, and the intraclass correlation coefficient (ICC) was calculated to enhance feature robustness and reproducibility (27). Furthermore, feature selection was initially conducted using logistic regression and subsequently refined through multivariate stepwise regression. A Pearson correlation heatmap was used to ensure selected features did not exhibit high collinearity. In addition, Shapley Additive Explanations (SHAP) analysis was used to improve the interpretability and transparency of the final models. Radiomics approaches have previously been applied to endometrial disease classification, generally showing strong performance for EC assessment. For example, Liu et al. constructed an MRI-based radiomics model and a visual nomogram, which effectively differentiated patients with benign and malignant endometrial lesions (26). However, the diagnostic accuracy of radiomics has been reported to be limited to 78.0% in test cohorts (26), and often suffer from limited generalizability and a lack of robustness across different datasets. Yan et al. developed a radiomics nomogram for fertility-preserving treatment candidate selection in EC and AEH, achieving an AUC of 0.94 in a prospective validation cohort-likely attributable to the reduced biases in prospective study (28).

In this study, the Clin+Rad model achieved higher AUC values than the DenseNet121-derived deep-feature model in the training and internal testing cohorts, suggesting that clinical information combined with radiomics features may provide complementary predictive value. However, this advantage was not consistently observed in the external validation cohort, where all models showed comparable performance. The discrepancy may reflect differences in cohort characteristics, sample size limitations, and potential center-specific variations. Notably, the age difference between AEH and EC patients was more pronounced in the training cohort (median age, 34 vs. 58 years), whereas this difference was smaller in the external validation cohort (50 vs. 55 years). Because age was an important clinical predictor, this inter-cohort difference may have introduced age-related selection bias and may partly explain the reduced external performance of the Clin+Rad model. Therefore, no single model can currently be considered definitively superior.

The DenseNet121-derived deep-feature model was trained using an axial slice with maximum tumor area from T2WI-FS, DWI, ADC, and late arterial phase CE-T1WI. Among these sequences, CE-T1WI and DWI provide essential functional information in the evaluation of EC and are frequently used as complementary tools to T2WI (27). Tumor enhancement patterns on CE-T1WI primarily reflect vascularity: although most EC are hypovascular, some lesions may appear iso- or hypervascular relative to the surrounding myometrium (29). In addition, DWI and ADC mainly reflect water diffusion characteristics, ADC values are negatively associated with tumor cell density (30), and prior studies have shown that ADC values in EC are significantly lower than those observed in AEH (31, 32). Studies have demonstrated that CNN-based methods achieved their highest diagnostic performance when trained on single axial ADC maps (27). Therefore, incorporating CE-T1WI, DWI, and ADC maps alongside T2WI facilitates a more accurate, comprehensive, and reliable diagnostic assessment of EC.

Given the strong diagnostic contribution of DWI and ADC-derived information discussed above, it is not surprising that a substantial proportion of discriminative features originated from the ADC maps in our study. This finding is clinically plausible, as EC typically demonstrates restricted diffusion which appears as hyperintensity relative to the surrounding myometrium on DWI and corresponding hypointensity on ADC maps (32). Previous studies have consistently underscored the value of ADC measurements in differentiating benign from malignant endometrial lesions (33, 34). For example, Tamai et al. reported that normal endometrium exhibited significantly higher ADC values compared with EC, supporting ADC as an initial imaging biomarker (32).

The robustness of ADC-derived radiomics features has also been validated across centers. In a multicenter study, Peerlings et al. reported that 122 ADC-based features demonstrated high reproducibility (ICC > 0.85) across different tissues, scanners, and vendors (35). This observation is consistent with our findings and explains the preferentially retention of ADC features during feature selection. Biologically, ADC values reflect the extent of water diffusion restriction, which becomes more pronounced in tumors with high cellularity and reduced extracellular space. A meta-analysis by Surov et al. confirmed a significant negative correlation between mean ADC values and tumor cellularity (36). Taken together, these biological and technical factors underscore the reliability and clinical relevance of ADC features in differentiating EC from AEH. Consistent with these findings, our study also showed that ADC-derived latent features constituted the predominant component of the final deep-feature set.

The DenseNet121-derived deep-feature model achieved the better predictive performance in the external validation cohort (AUC, 0.854), representing a notable improvement over the Radiomics model (AUC 0.803) and demonstrating its superior generalizability. As outlined earlier, numerous DL architectures have been successfully applied to medical imaging tasks. For example, Takahashi et al. developed a DL algorithm trained on more than 411,800 hysteroscopic images, achieving strong discriminatory capacity across five endometrial disease categories (24). Other work has applied DL models to pathological image, with high PPV (82.94%) and NPV (96.53%) both demonstrating strong accuracy (37). In contrast, the NPV of all models in our study was substantially lower than the PPV. This discrepancy may be attributable to sample imbalance, as positive cases were more prevalent—particularly in the external validation cohort, where EC cases was twice that of AEH cases. To date, few studies MRI-based DL model has been reported for differentiating EC from AEH, highlighting the novelty and clinical significance of our work.

There are several limitations to this study. First, the relatively small sample size, particularly in the external validation cohort, and the lack of prospective validation may limit the generalizability of our findings. Second, manual lesion segmentation is time-consuming and susceptible to inter-observer variability, and automatic or semi-automatic segmentation approaches may improve efficiency and reproducibility. Third, although the sequential feature-selection strategy combining LASSO and stepwise logistic regression was used to reduce feature redundancy and improve interpretability, it may introduce additional selection variability. Fourth, conventional radiologist interpretation and direct radiologist-versus-model comparisons were not prospectively incorporated into the study protocol, limiting assessment of the model’s added value over routine clinical interpretation. In addition, the absence of hysterectomy confirmation or longitudinal follow-up in some patients with AEH may have resulted in potential misclassification, as occult concurrent endometrial carcinoma cannot be completely excluded. Therefore, the proposed model should be considered an adjunctive tool rather than a replacement for pathological diagnosis, with potential applications in post-diagnostic risk stratification, identification of AEH patients at increased risk of concurrent stage I EC, and support for decisions regarding additional sampling or surgical planning. Future prospective multicenter studies with larger and more heterogeneous cohorts are warranted to further evaluate model robustness, external generalizability, feature stability, potential recalibration strategies, and the clinical value of automated segmentation and radiologist-versus-model comparisons.

## Conclusions

In this study, we developed an MRI-based deep feature model to assist in the preoperative differentiation of stage I EC from AEH. The DenseNet121-derived deep-feature model showed comparable diagnostic performance with the Radiomics and Clin+Rad models and relatively stable performance across the internal and external cohorts. Using a probability threshold of 0.563 determined in the training cohort by the Youden index, the model provided quantitative estimates of the probability of stage I EC and may serve as an adjunctive tool to support radiologists in preoperative risk assessment and clinical decision-making regarding further invasive management. Given the limited sample size of the external validation cohort and the absence of statistically significant differences in pairwise AUC comparisons, these findings should be considered preliminary and require validation in larger, prospective multicenter cohorts.

## Data Availability

The raw data supporting the conclusions of this article will be made available by the authors, without undue reservation.
