# Supplementary material for: MRI-derived deep-feature model for differentiating stage I endometrial carcinoma from atypical endometrial hyperplasia: a two-center retrospective study
Source: Front Oncol. 2026 Sep 14;16:1943943. doi: 10.3389/fonc.2026.1943943 (PMC13616772; doi:10.3389/fonc.2026.1943943)
Supplement: Supplementary file 1 [file DataSheet1.pdf]

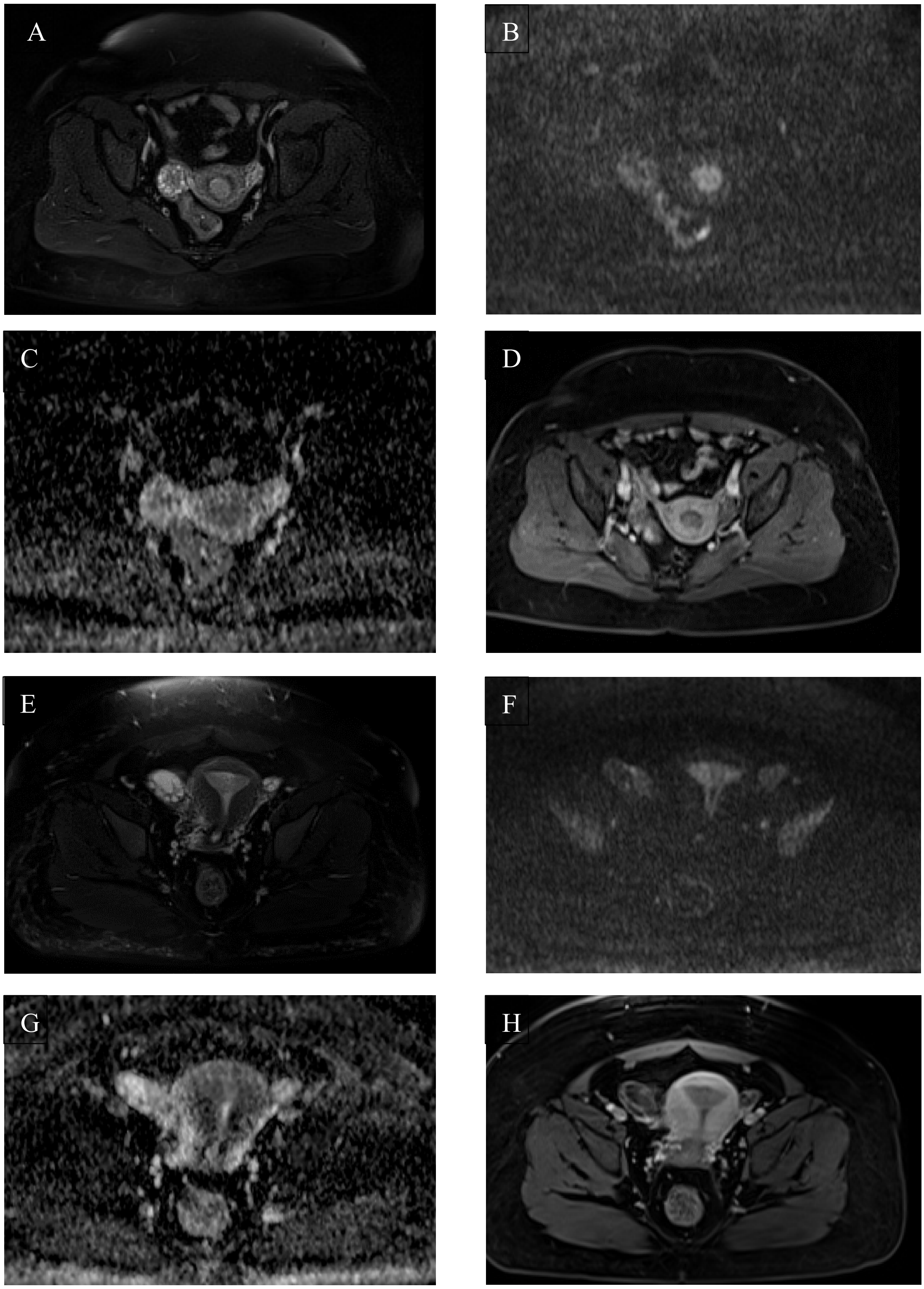

Supplementary Figures S1: Representative T2WI-FS, DWI, ADC, and CE-T1WI images of EC and AEH.

An EC patient (A–D) showed a round intrauterine mass with intermediate signal intensity on T2WI-FS, marked hyperintensity on DWI, marked hypointensity on the

ADC map, and mild heterogeneous enhancement on CE-T1WI acquired approximately 90 s after contrast administration. The posterior endometrial–myometrial junction was indistinct, suggesting myometrial invasion. An AEH patient (E–H) showed diffuse endometrial thickening with slightly low signal intensity on T2WI-FS, mildly increased signal intensity on DWI, slight hypointensity on the ADC map, and mild heterogeneous enhancement on CE-T1WI acquired approximately 90 s after contrast administration.

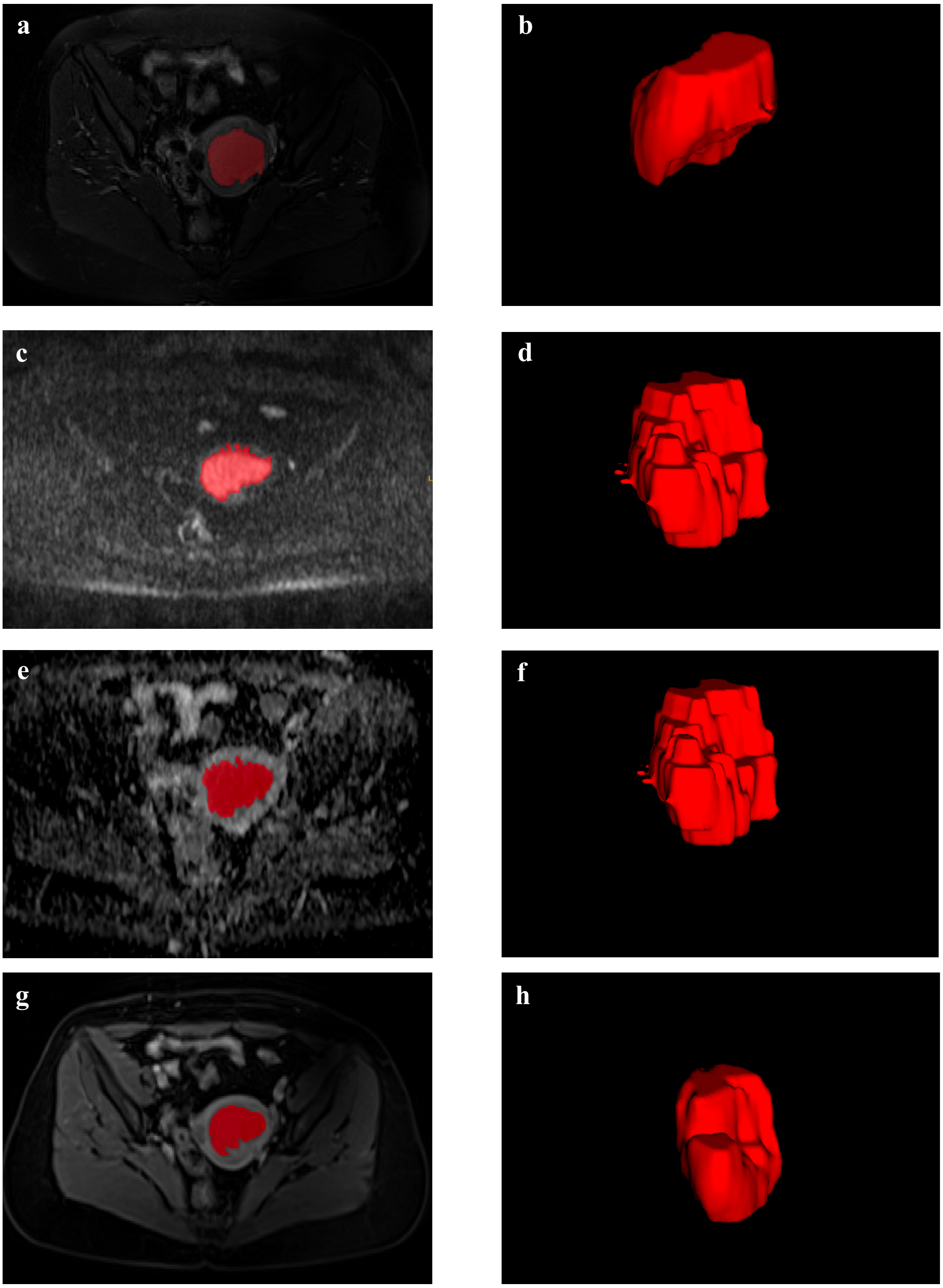

Supplementary Figure S2：Schematic illustration of ROI delineation on T2WI-FS, DWI, ADC, and CE-T1WI.

a, c, e, and g show the ROIs delineated on T2WI-FS, DWI, ADC, and CE-T1WI images, respectively; whereas panels b, d, f, and h show the corresponding three-dimensional volumes of interest (VOIs).

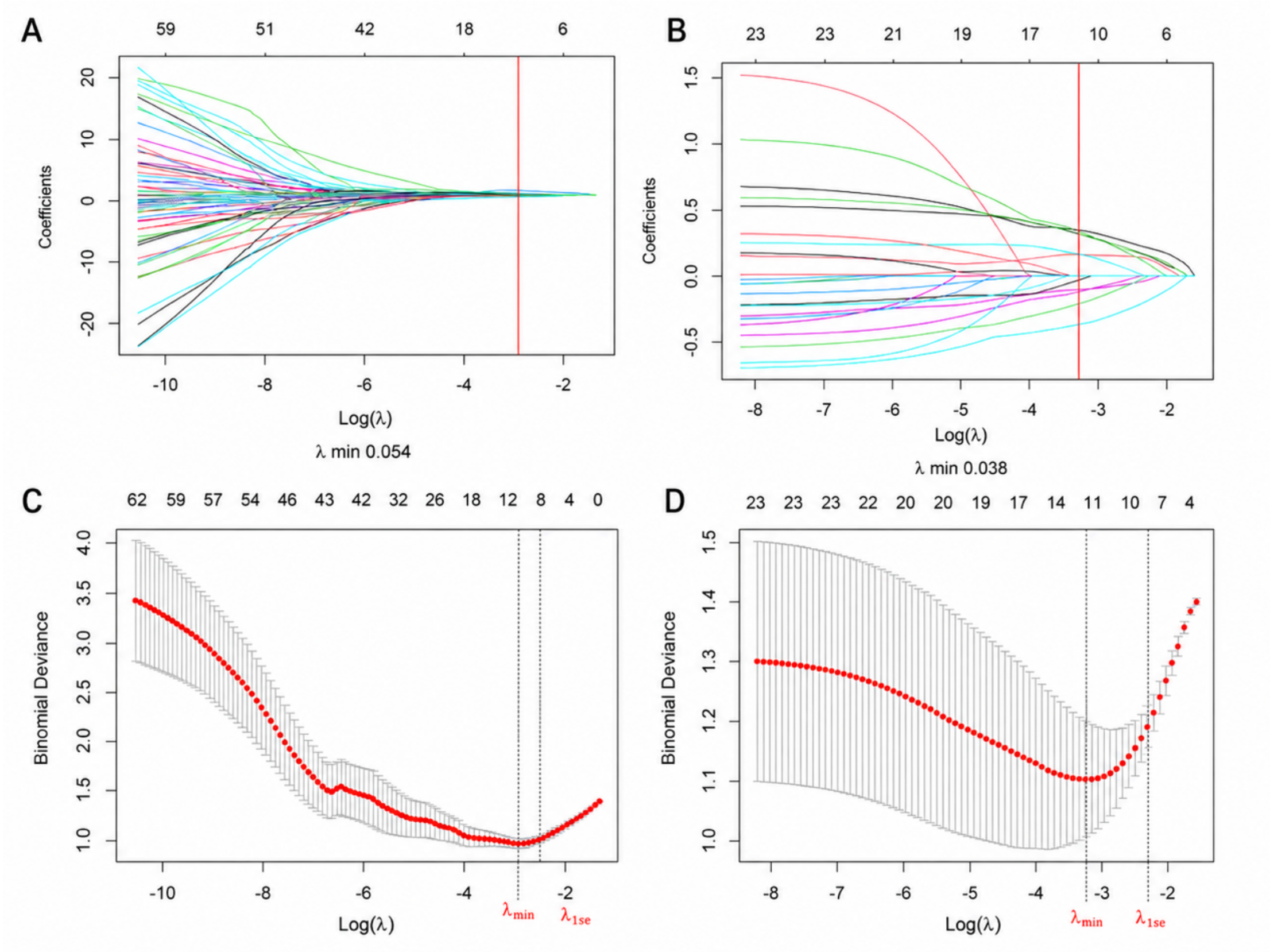

Supplementary Figure S2. LASSO feature selection for radiomics and deep features.

(A, C**)** LASSO feature-selection results for the Radiomics model. **(**B, D) LASSO feature-selection results for the DenseNet121-2D deep-feature model. (A-B) The coefficient paths demonstrate the effect of LASSO regularization on feature selection. As the regularization parameter λ (log λ) increases, the absolute values of more coefficients shrink toward zero, leading to the removal of redundant or weakly contributing features. (C, D) Cross-validation curves showing the relationship between the regularization parameter and binomial deviance. The binomial deviance initially decreases with increasing λ, indicating improved model fit, and subsequently increases when λ becomes excessively large because of over-regularization. The λ corresponding to the minimum cross-validated deviance (λ min) was selected. (LASSO, least absolute shrinkage and selection operator.)

Supplementary Table S1. Detailed PyRadiomics feature extraction parameters

Parameter	Setting
Software	PyRadiomics 2.2.0
Image types	Original, Wavelet, LoG, SquareRoot, Logarithm, Exponential, Gradient, LBP
Feature classes	First-order, shape, texture, filtered-image features
Bin width	25
T2WI discretization	5 gray levels
DWI discretization	5 gray levels
CE-T1WI discretization	5 gray levels
ADC discretization	50 gray levels
Resampling voxel size	$1.5 \times 1.5 \times 1.5$ mm^3
Image interpolation	B-spline interpolation
Mask interpolation	Nearest-neighbor interpolation
Intensity normalization	Z-score normalization
Wavelet filtering	8 wavelet decompositions
LoG filtering	Applied
SquareRoot filtering	Applied
Logarithm filtering	Applied
Exponential filtering	Applied
Gradient filtering	Applied
LBP filtering	LBP3D; three parameter settings
IBSI compliance	Yes

Supplementary Table S2. Regression coefficients, odds ratios, 95% confidence intervals, P values, and complete equations for the Radiomics, DenseNet121-derived deep-feature, Clinical, and Clin+Rad models.

1.Radiomics

Feature	coef	p	OR (95% CI)
**(Intercept)**	0.049	0.828	
**ADC_wavelet.HLL_firstorder_Median**	0.652	0.015	1.919(1.141-3.419)
**ADC_wavelet.LHL_firstorder_Skewness**	-0.572	0.054	0.564(0.305-0.982)
**ADC_wavelet.LHL_firstorder_Median**	1.529	< 0.001	4.614(2.178-10.686)
**T2WIFS_lbp.3D.m1_glcm_JointEnergy**	-0.733	0.008	0.480(0.270-0.795)
**T2WIFS_log.5_glcm_Imc2**	0.621	0.0158	1.860(1.158-3.213)
**ADC_original_glszm_LowGrayLevelZone Emphasis**	-0.358	0.112	0.699(0.431-1.061)
**ADC_gradient_glcm_Imc2**	0.371	0.100	1.449(0.935-2.280)

η_Rad = 0.049
+ 0.652 × ADC_wavelet.HLL_firstorder_Median
- 0.572 × ADC_wavelet.LHL_firstorder_Skewness
+ 1.529 × ADC_wavelet.LHL_firstorder_Median
- 0.733 × T2WIFS_lbp.3D.m1_glcm_JointEnergy
+ 0.621 × T2WIFS_log.5_glcm_Imc2
- 0.358 × ADC_original_glszm_LowGrayLevelZoneEmphasis
+ 0.371 × ADC_gradient_glcm_Imc2
prediction probability

P_Rad (Y = 1) = 1 / [1 + exp(-η_Rad)]

2.DenseNet121-derived deep-feature model

Feature	coef	p	OR (95% CI)
**(Intercept)**	0.100	0.632	
**ADC_F16**	0.489	0.065	1.630(0.983-2.806)
**ADC_F961**	0.624	0.016	1.866(1.150-3.193)
**DWI_F253**	-0.642	0.004	0.526(0.332-0.801)
**ADC_F502**	0.559	0.030	1.749(1.066-2.947)
**ADC_F482**	-0.358	0.104	0.699(0.449-1.075)
**T2WIFS_F87**	0.604	0.016	1.830(1.146-3.100)
**T2WIFS_F572**	-0.465	0.057	0.628(0.378-0.992)
**ADC_F477**	0.409	0.057	1.505(0.999-2.327)

η_DL = 0.100329
+ 0.489 × ADC_F16
+ 0.624 × ADC_F961
- 0.642 × DWI_F253
+ 0.559 × ADC_F502
- 0.358 × ADC_F482
+ 0.604 × T2WIFS_F87
- 0.465 × T2WIFS_F572

+ 0.409 × ADC_F477
prediction probability

P_DL (Y = 1) = 1 / [1 + exp(-η_DL)]

3.Clinical model

Feature	coef	p	OR
**Clinical (Intercept)**	-6.754	< 0.001	
**Age**	0.136	< 0.001	1.146(1.078-1.229)
**Menopause1**	2.229	0.015	9.287(1.790-73.613)

η_Clinical = -6.753688

+ 0.136458 × Age

+ 2.228661 × Menopause1

prediction probability

P_Clinical(Y = 1) = 1 / [1 + exp(-η_Clinical)]

3.Clin+Rad model

Feature	coef	p	OR
**(Intercept)**	-7.911	3.402	
**Age**	0.173	< 0.001	1.189(1.093-1.318)
**Menopause1**	2.461	0.051	11.721(1.171-193.241)
**ADC_wavelet.HLL_firstorder_Median**	1.087	0.005	2.967(1.384-6.711)
**ADC_wavelet.LHL_firstorder_Median**	1.557	0.002	4.744(1.883-14.486)
**T2WIFS_lbp.3D.m1_glcm_JointEnergy**	-0.994	0.040	0.370(0.132-0.899)
**ADC_original_glszm_Low GrayLevelZoneEmphasis**	-0.836	0.056	0.433(0.163-0.903)

η_Clin+Rad = -7.910798

+ 0.173202 × Age

+ 2.461369 × Menopause1

+ 1.087441 × ADC_wavelet.HLL_firstorder_Median

+ 1.556907 × ADC_wavelet.LHL_firstorder_Median

- 0.993913 × T2WIFS_lbp.3D.m1_glcm_JointEnergy

- 0.836007 × ADC_original_glszm_LowGrayLevelZoneEmphasis

prediction probability

P_Clin+Rad(Y = 1) = 1 / [1 + exp(-η_Clin+Rad)]

Supplementary Table S3：Comparison of NRI and IDI for Incremental Diagnostic Performance among Different Models across Different Cohorts

Different model	Cohort	NRI (95% CI)	P value	IDI (95% CI)	P value
DenseNet121-derived vs Radiomics	Training	-0.079 (-0.254, 0.097)	0.380	-0.092 (-0.172, -0.011)	0.025
	Internal Testing	-0.349 (-0.627, -0.070)	0.014	-0.186 (-0.324, -0.049)	0.008
	External Validation	0.227 (-0.023, 0.477)	0.075	0.135 (0.019, 0.250)	0.022
DenseNet121-derived vs Clin+Rad	Training	-0.374 (-0.538, -0.210)	<0.001	-0.323 (-0.406, -0.239)	<0.001
	Internal Testing	-0.721 (-0.983, -0.459)	<0.001	-0.453 (-0.612, -0.294)	<0.001
	External Validation	0.059 (-0.262, 0.380)	0.719	0.007 (-0.196, 0.211)	0.943
Radiomics vs Clin+Rad	Training	0.351 (0.216, 0.488)	<0.001	0.231 (0.170, 0.291)	<0.001
	Internal Testing	0.426 (0.201, 0.651)	<0.001	0.267 (0.147, 0.386)	<0.001
	External Validation	0.099 (-0.179, 0.378)	0.484	0.128 (-0.047, 0.302)	0.151

Note: NRI and IDI were used to assess the incremental diagnostic value of the comparison model relative to the reference model. NRI and IDI additionally reflect changes in case reclassification and overall discriminative ability, and may therefore complement AUC-based comparisons. NRI/IDI > 0 indicates that the former model outperforms the latter in classification or discrimination ability, whereas NRI/IDI < 0 indicates poorer performance of the former model relative to the latter.

(95% CI: 95% confidence Interval; NRI: Net Reclassification Improvement; IDI: Integrated Discrimination Improvement;)
